# Species diversity and phylogeography of the *Australoheros autrani* group (Teleostei, Cichlidae) in eastern Brazil

**DOI:** 10.1007/s10228-022-00888-9

**Published:** 2022-09-27

**Authors:** Marcos A. da Silva, Felipe P. Ottoni, José L. O. Mattos, Adrian Indermaur, Axel M. Katz, Walter Salzburger

**Affiliations:** 1 Zoological Institute, Department of Environmental Sciences, Universität Basel, Basel, Switzerland; 2 Laboratório de Sistemática e Ecologia de Organismos Aquáticos, Centro de Ciências Agrárias e Ambientais, Universidade Federal do Maranhão, Chapadinha, Brazil; 3 Laboratório de Sistemática e Evolução de Peixes Teleósteos, Universidade Federal do Rio de Janeiro, Rio de Janeiro, Brazil

**Keywords:** Heroini, Diversification, Neotropical region, Paraná River basin, Phylogeography

## Abstract

Cichlid fishes are an important model system in evolutionary biology, primarily because of their exceptional diversity. However, while some cichlid assemblages, such as the ones of the African Great Lakes, have received considerable attention, others are not well studied, including many riverine species. Here, we focus on the *Australoheros autrani* species group and first report a new record of *Australoheros* in the upper Paranaíba River drainage, extending the known distribution range of this genus. Through Bayesian inference and maximum likelihood phylogenetic analyses of the mitochondrial cytochrome *b* gene of these specimens as well as available sequences, we assigned the newly discovered population to *Australoheros barbosae*. We corroborate the monophyly of the *A. autrani* species group and the presence of three species in the upper/middle Paraíba do Sul River basin as well as molecular diagnostic characters for each. Finally, we provide evidence for a recent expansion of *A. barbosae*.

**Supplementary Information:**

The online version contains supplementary material available at 10.1007/s10228-022-00888-9.

## Introduction

Cichlid fishes (Cichlidae) are one of the most taxonomically, morphologically and ecologically diverse families of fishes and renowned as an important model system and textbook example in evolutionary biology (Kocher 2004; Salzburger 2018). First and foremost, the spectacular adaptive radiations of cichlid fishes in the African Great Lakes Victoria, Malawi and Tanganyika have received considerable scientific attention (Meier et al. 2017; Malinsky et al. 2018; Ronco et al. 2021). Cichlids are, however, much more widely distributed than in East Africa and occur in large parts of tropical and subtropical Africa, the Americas, India and Madagascar (see e.g., Matschiner et al. 2020), whereby especially riverine cichlid faunas are typically much less explored in terms of taxonomy, ecology and evolution compared to the ones of lakes.

*Australoheros* is a South American cichlid genus in the tribe Heroini (Ottoni et al. 2019). The genus comprises 28 valid species, excluding *Heros autochthon*. This latter species has previously been placed within *Australoheros*; however, there are a number of doubts and problems regarding its type locality, the type material and the original description, making it impossible to link it to any known lineage (or species) of the genus (Ottoni and Bragança 2021). *Australoheros* shows a wide distribution range, with representatives occurring in river basins and systems from north-eastern Argentina and Uruguay to the South of the state of Bahia in eastern Brazil (Casciotta et al. 1995; Říčan and Kullander 2003, 2008; Ottoni and Costa 2008; Ottoni 2010; Ottoni et al. 2011, 2019; Říčan et al. 2011).

The genus *Australoheros* has previously been divided into five species groups: (*i*) the *forquilha* group, (*ii*) the *scitulus* group, (*iii*) the *kaaygua* group, (*iv*) the *facetus* group [these four groups were proposed by Říčan and Kullander (2008) based on morphological and molecular (mitochondrial cytochrome *b*; cyt *b*) information], and (*v*) the *autrani* group (or: *Australoheros autrani* species group), which was proposed by Ottoni (2010, 2011, 2012) based on morphological characters. The monophyly of this latter species group has subsequently been corroborated by means of phylogenetic analyses of cyt *b* sequences (Ottoni et al. 2019). The *autrani* group consists of three clades with a non-overlapping geographic distribution range (Ottoni et al. 2019): first, a clade found in the upper/middle Paraíba do Sul River basin and adjacent drainages including Rio Macacu, the upper Rio Tietê, the Rio Grande, the upper Rio Paraná, the upper Rio Paraopebas, the Rio das Velhas, tributaries of the upper Rio São Francisco, as well as the headwaters between the Rio Doce and Rio São Francisco in south-eastern Brazil; second, the Northern Mata Atlântica clade, which is distributed along the coastal river basins from the Saquarema lagoon system in south-eastern Brazil to the Buranhém River basin in north-eastern Brazil, and including tributaries of the lower Rio Paraíba do Sul and the Rio Doce basin; third, the Southern Mata Atlântica clade, occurring in the Rio Ribeira do Iguape basin and in the Rio Cubatão basin of the Baía de Babitonga system in eastern Brazil (Ottoni et al. 2019).

Here, we report a new record of *Australoheros* in the upper Paranaíba River drainage, of the upper Paraná River basin, eastern Brazil, substantially extending the known distribution range of this genus. We then evaluate the phylogenetic position of these new specimens through a phylogeographic analysis of all available cyt *b* sequences and conduct a species delimitation analysis, focusing on the *Australoheros autrani* species group.

## Materials and methods

**Fig. 1:**
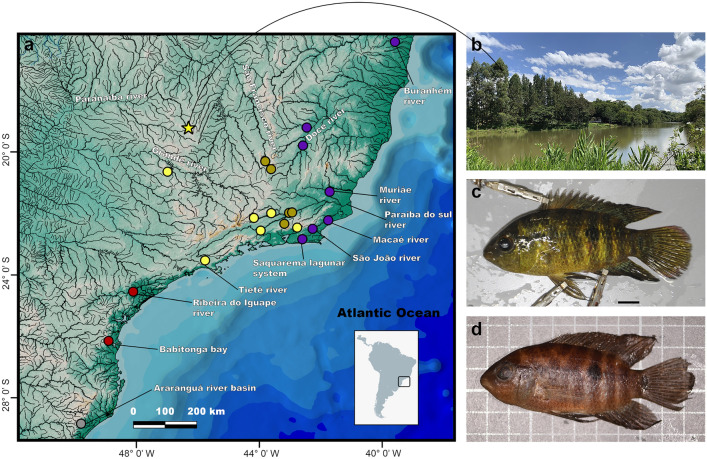
**a** Map of the in-group samples of the present study. *Circles* and *star*, *Australoheros autrani* species group. *Yellow circles*, UMPSAD clade; *yellow star*, six recently collected specimens (collected in October 2019, see Table 1); *red circles*, Southern Mata Atlântica clade; *blue circles*, Northern Mata Atlântica clade; *gray circle*, *A.* sp. Timbé do Sul (geographic closest out-group). **b** Sampling site for the new population of *Australoheros* in the Rio Paranaíba River basin. Dammed stream (Olhos D’Água stream) in a farm (Fazenda 3 Barras), 5 km from the town of Rio Paranaíba, State of Minas Gerais, Brazil. **c** Collected specimen of *Australoheros* from the upper Paranaíba River Drainage, minutes after killing (Voucher number LAGEEVO 4374); scale bar = 1 cm. **d** Same specimen fixed

*Sampling and processing.* New specimens of *Australoheros* were collected in October 2019 in the upper Paranaíba River drainage of the upper Paraná River basin in eastern Brazil (Fig. 1), a river drainage from which no records for the genus *Australoheros* existed so far. Specimens were measured, weighed, and photographed (Fig. 1c); a fin clip of each specimen was taken and fixed in 96% ethanol immediately after collection as DNA sample, and whole specimens were fixed in 10% formaldehyde for three days and later stored in 70% ethanol. The voucher specimens were deposited in the fish collection of the Laboratório de Genética Ecológica e Evolutiva at the Universidade Federal de Viçosa, Campus Rio Paranaíba (LAGEEVO, UFV-CRP). Additional material was taken from a previous study (Ottoni et al. 2019), and for each focal species and population, we used samples taken at different localities within the distribution range of the *Australoheros autrani* species group, and taxonomically identified the species according to Ottoni et al. (2019) (see Fig. 1 and Table 1 for further details on the specimens used in this study as well as their sampling localities).

**Table 1:** Haplotypes of *Australoheros* sp*.* sampled in the Paranaíba River basin

Voucher number	Collection location and river basin	Coordinates	Collection date	Collected by	GenBank accession number
LAGEEVO 4365	Olhos D’Água stream, Fazenda 3 Barras, municipality of Rio Paranaíba, State of Minas Gerais, Brazil/Paranaíba River drainage of the upper Paraná River basin	−19.22212574, −46.30496552	18 October 2019	Marcos Aurélio da Silva, Iuri Silva, Orlando da Silva	OM327755
LAGEEVO 4367					OM327756
LAGEEVO 4368					OM327757
LAGEEVO 4369					OM327758
LAGEEVO 4373					OM327759
LAGEEVO 4374					OM327760

*DNA extraction, amplification, sequencing and alignment.* Genomic DNA was extracted from ethanol-preserved fin clips using the DNeasy Blood and Tissue Kit (Qiagen) and following the manufacturer’s protocol. For polymerase chain reaction (PCR) amplification of a 1,039 bp-long fragment of the mitochondrial cyt *b* gene, we used the primers CytB-F (Palumbi et al. 1991) and TrucCytB-R (Martin and Bermingham 1998). PCR was performed in a 50 µl master reaction mixture containing 10× Taq buffer (Biozyme), 8.25 μl of ddH2O, 1 μM of each primer, 75 ng of total genomic DNA, 0.2 mM of each dNTP, and 1 U of RedTaq polymerase (Biozyme). The thermocycling profile on a Veriti 96-well thermal cycler (Applied Biosystems) was 35 cycles of 30 s at 94°C, 30 s at 50°C and 1 min at 72°C, followed by 3 min at 72°C. Negative controls were used in all experiments. Amplified PCR products were then purified using the GenElute Gel Extraction Kit (Sigma-Aldrich). The sequencing PCR was performed in reactions containing 1 µl of BigDye 3.1 reaction mix (ThermoFisher Scientific), 0.5 µl of primer solution and 6.5 µl of purified DNA. The thermocycling profile on a Veriti 96-well cycler was 1 cycle of 1 min at 96°C, 25 cycles of 10 s at 96°C, 20 s at 52°C, and 2 min and 30 s at 60°C, and a resting cycle at 4°C. Reactions were purified with the X-Terminator mix containing 15 µl of ddH2O, 22.5 µl of SAM solution and 5 µl of X-Term Bead centrifuged at 2,250 rpm for 30 min. Sanger sequencing was performed on an ABI 3130*xl* Genetic Analyser (Applied Biosystems). Sequences were inspected by eye, and the forward and reverse reads were edited and aligned with CodonCode Aligner version 2.0.6 (CodonCode Corporation).

*Phylogenetic analyses and mitochondrial mismatch analysis.* To obtain phylogenetic hypotheses for the *Australoheros autrani* group based on the new and available cyt *b* sequences, we performed maximum likelihood (ML) and Bayesian inference (BI) analyses, partitioning the protein-coding *cyt b* according to codon position. We used PartitionFinder 2.1.1 (Lanfear et al. 2016) to identify the most appropriate substitution model based on the Bayesian information criterion (BIC; Schwarz 1978): K80 + I for first codon positions, HKY for second codon positions and TRN + G for third codon positions. A maximum likelihood (ML) phylogenetic analysis was performed with IQtree (Chernomor et al. 2016) in the ultrafast bootstrap mode (UFBoot) (Hoang et al. 2018) to assess the congruence between different tree search algorithms and statistical methods. Bayesian inference was performed with MrBayes 3.2.7 (Huelsenbeck and Ronquist 2001; Ronquist and Huelsenbeck 2003), running two Markov chain Monte Carlo (MCMC) simulations of four chains each for 50 million generations, with a sampling frequency of 1,000. All parameters between partitions, except topology and branch lengths, were unlinked. The convergence of the MCMC chains was assessed by evaluating the stationary phase of the chains using Tracer v. 1.7.1 (Rambaut et al. 2018. A consensus topology and posterior probabilities were obtained after applying a burn-in of 25%. Finally, we produced a haplotype genealogy with Fitchi (Matschiner 2015), based on the transformation of a bifurcating phylogenetic tree in haplotype genealogies as described in Salzburger et al. (2011).

**Fig. 2:**
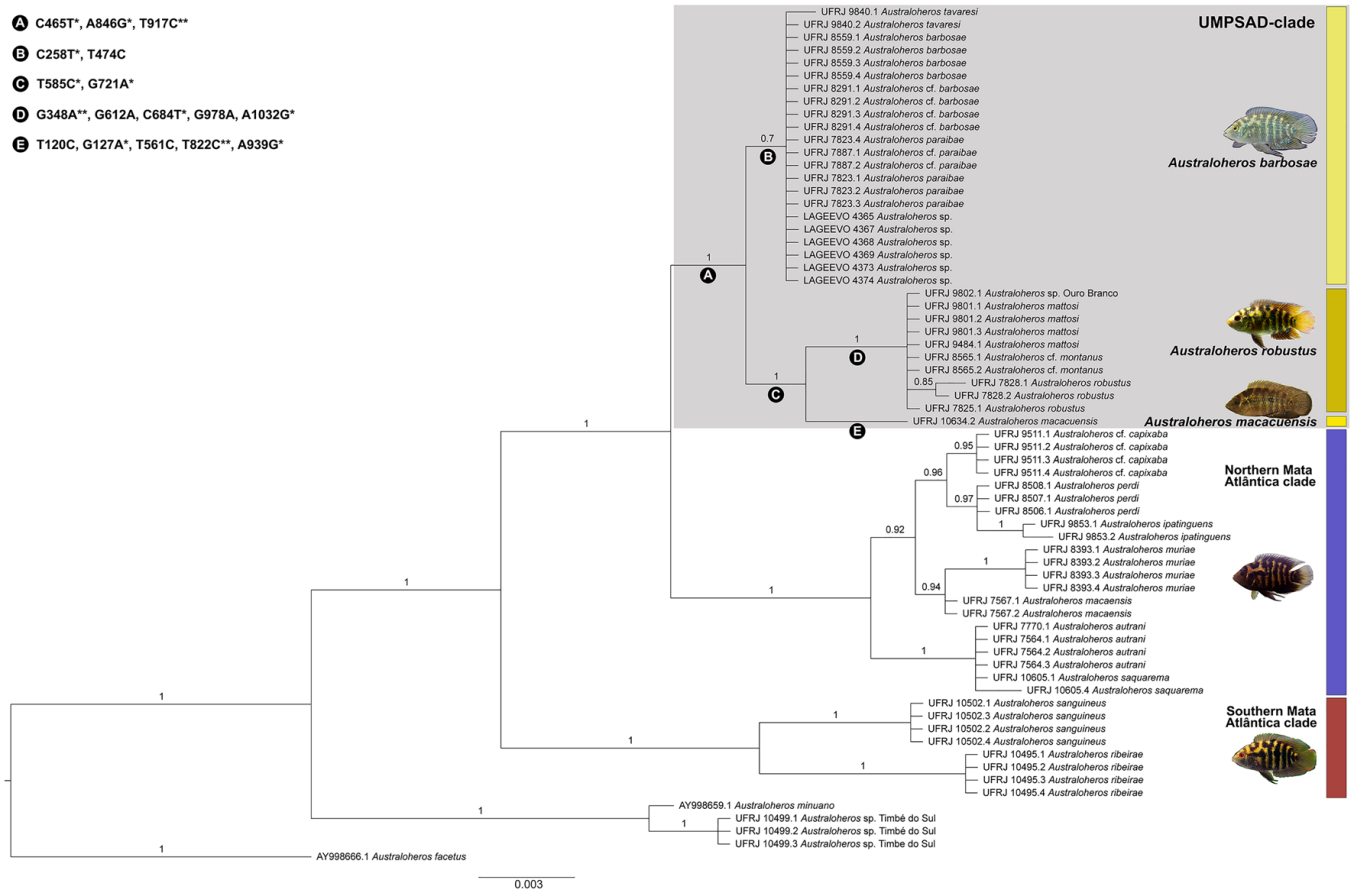
Bayesian inference (BI) tree of *Australoheros* from south-east Brazil. The in-group of the present work (UMPSAD clade) is highlighted in light gray. Numbers above branches are posterior probability values. Letters below branches represent the combination of nucleotide substitutions (only for the in-group taxa) that define the species (in CBB) or clades. A list of nucleotide substitutions is provided in the upper left. When a nucleotide substitution is exclusive from that lineage or clade, not occurring in any other branch, it is marked with “*”; when a nucleotide substitution is exclusive within the *A. autrani* group it is marked with “**”. Species of the in-group delimited through the tree-based method (WP) are indicated with vertical yellow bars

We then calculated the mismatch distribution as the observed pairwise nucleotide site differences (Watterson 1975; Rogers and Harpending 1992) to compare the demographic history of the major evolutionary lineages in our dataset, that is, the ‘Southern Mata Atlântica’, the ‘Northern Mata Atlântica’ and the ‘UMPSAD’ clade as well as the focal species *Australoheros barbosae* and *Australoheros robustus* (see Fig. 2). For this analysis, we used the software DnaSP version 6.12.03 (Rozas et al. 2017) and estimated Tajima’s *D* (Tajima 1989), Ramos-Onsins and Rozas’s *R2*, and the raggedness statistic, *r* (Harpending 1994). We could not perform such analyses for *A. robustus*, as we only had one sequence for this species.

*Species delimitation.* Two distinct strategies for species delimitation were used as operational criteria (both based on the molecular information provided by cyt *b*): a tree-based method as proposed by Wiens and Penkrot (2002) [hereafter called WP, following Sites and Marshall (2003)] and a character-based DNA barcoding method as proposed by Desalle et al. (2005) (hereafter called CBB) and adapted by Ottoni et al. (2019).

The terminal in-group taxa for species delineation were the *a priori* defined species and populations from the upper/middle Paraíba do Sul River basin and adjacent drainages (Ottoni et al. 2019), hereafter called UMPSAD clade. Terminal out-group taxa included several lineages representing all the other clades of the *Australoheros autrani* group, as well as an additional population from the south of the State of Santa Catarina (*Australoheros* sp. “Timbé do Sul”), *Australoheros minuano* and *Australoheros facetus*, which were considered the most closely related species to the *Australoheros autrani* group according to Ottoni et al. (2019). The criteria to evaluate the topology and its support followed Wiens and Penkrot (2002) and Ottoni et al. (2019).

For the WP species delimitation method, we used the Bayesian inference phylogeny (Fig. 2). The CBB method was performed through optimization of nucleotide substitutions among lineages of *Australoheros* obtained from the Bayesian topology using PAUP*4 (Swofford and Sullivan, 2003). Here, each nucleotide substitution is represented by its relative numeric position determined through sequence alignment with the complete mitochondrial genome of *Astronotus ocellatus* (Mabuchi et al. 2007). We considered as valid only the species of the in-group corroborated by both species delimitation methods applied here.

## Results

**A new record for *Australoheros* in the Paranaíba River basin.** We here provide the first record of a species of *Australoheros* in the Paranaíba River drainage. Six specimens of *Australoheros*, assigned to *A. barbosae*, were sampled in October 2019 (see Table 1 for further details) close to the source of the Olhos D’Água stream, one of the tributaries of the Paranaíba River, where private dams in farmlands are abundant, potentially affecting the native ichthyofauna, with only one other taxon being present, the characin *Astyanax lacustris*. We thus substantially expand the known distribution range of the *Australoheros autrani* species group (more specifically *A. barbosae*) to the Paranaíba River drainage (Fig. 1a).

**Fig. 3:**
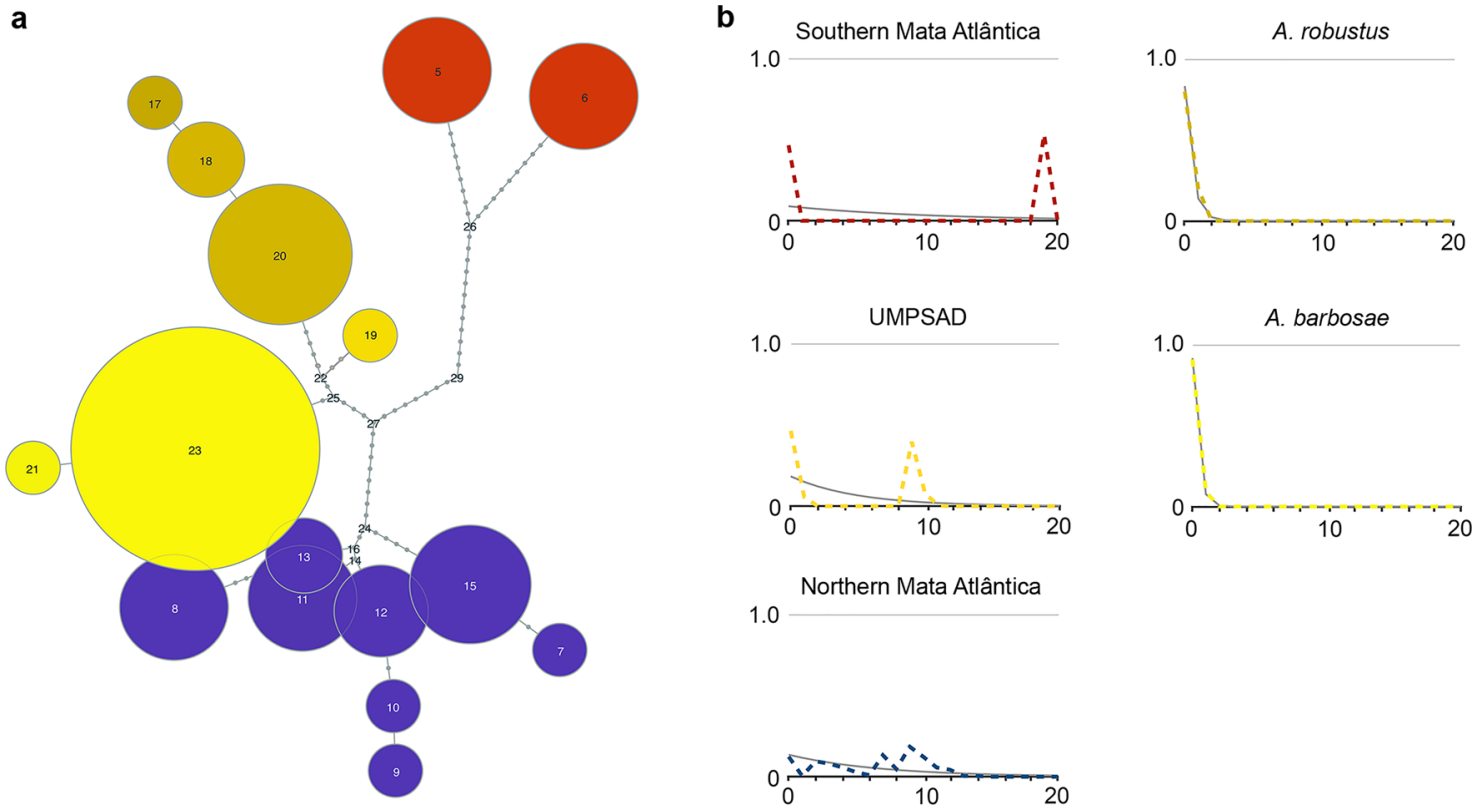
**a** Haplotype genealogy of the *Australoheros autrani* group. Node size represents the number of sequence records and edge length represents the number of substitutions (transitions or transversions). **b** Results from the mismatch analyses. The x-axis indicates the number of mutational differences (from 0 to 20), and the y-axis shows the relative number of pairwise distances (observed frequency in dashed line, expected frequency in gray color). The color scheme refers to Fig. 1

**Mitochondrial phylogeny and haplotype genealogy of the *Australoheros autrani* species group.** The BI and ML phylogenetic trees were highly congruent [Fig. 2; Electronic Supplementary Material (ESM) S1]. The *Australoheros autrani* species group and the clade containing the representatives of the upper/middle Paraíba do Sul River basin and adjacent drainages were recovered as monophyletic, with maximum node support values (Fig. 2). The UMPSAD clade was diagnosed by three exclusive nucleotide substitutions, as indicated by “A” in Fig. 2. The representatives of *A. barbosae* were recovered as being monophyletic as well, as sister clade to *Australoheros macacuensis* and *A. robustus*, with a node support of 0.7 posterior probability (Fig. 2). The new sequences from the Olhos D'Água stream in the Alto Paranaíba drainage clustered with a known *A. barbosae* haplotype (Fig. 3a). There was no shared mitochondrial haplotype between species. The nucleotide diversity is summarized in Table 2.

**Table 2:** Nucleotide diversity of the *Australoheros autrani* group

All *Australoheros minuano* *Australoheros facetus* *Australoheros barbosae* *Australoheros robustus* Northern MA clade Southern MA clade *Australoheros macacuensis*	123 0 0 1 2 19 19 0	916 1,039 1,039 1,038 1,037 1,020 1,020 1,039	0.1184 0.0000 0.0000 0.0010 0.0019 0.0183 0.0183 0.0000	0.0204 0.0000 0.0000 0.0001 0.0006 0.0061 0.0104 0.0000

**Demographic analyses.** This mismatch analysis (Fig. 3b; ESM S2) revealed similar distributions of the pairwise nucleotide site differences in the ‘Northern Mata Atlântica’ and the ‘UMPSAD’ clade, with a first peak around 10 differences, suggesting that the initial expansion of these clades occurred roughly around the same time. In contrast, the ‘Southern Mata Atlântica’ clade showed a first peak at around 20 mutations, suggesting—similar to the phylogenetic analyses—an older age of this clade. The two focal species *A. barbosae* and *A. robustus* show a distribution that is compatible with a very recent expansion (Fig. 3b), as also indicated by negative Tajima’s *D* values of -1.16 and -1.11, respectively (ESM S2).

**Species delimitation.** Both approaches (WP and CBB) led to identical results, delimiting three lineages (species) within the upper/middle Paraíba do Sul River basin and adjacent drainages clade (see box in Fig. 2): *Australoheros barbosae*, *A. macacuensis*, and *A. robustus* (see also Ottoni 2013; Ottoni and Schindler 2014; Ottoni et al. 2014 for additional diagnostic features proposed after the original descriptions of the three species). Despite the low node support (posterior probably lower than 0.95) for the monophyly of *A. barbosae*, this species was delimited by the WP method since its sister group was well supported (node support higher than 0.95 of posterior probability value).

The species *A. macacuensis* and *A. robustus* were recovered as sister species, sharing two diagnostic and exclusive nucleotide substitutions (“C” in Fig. 2). *Australoheros robustus* was recovered as an exclusive species, with maximum node support and five diagnostic nucleotide substitutions (three of which were exclusives) (“D” in Fig. 2). *Australoheros macacuensis* was diagnosed by five nucleotide substitutions (three of them being exclusive) (“E” in Fig. 2).

Although *A. macacuensis* was represented in our dataset by a single haplotype, it was delimited as a separate lineage (species) by the WP method, because its sister species received maximum support. *Australoheros barbosae* was recovered as separate species by two diagnostic nucleotide substitutions, of which one was exclusive (“B” in Fig. 2). The combined results of these species delimitations methods, as well as the taxonomic status suggested from those, are summarized in ESM S3.

## Discussion

In this study, we report the finding of specimens of the cichlid genus *Australoheros* in the upper Paranaíba River drainage (Fig. 1), thus substantially extending the distribution range of this genus. Through phylogenetic analyses based on their mitochondrial cyt *b* gene and available sequences, we could assign these specimens to *A. barbosae.*

Our phylogenetic analyses based on maximum likelihood and Bayesian inference support the monophyly of the *Australoheros autrani* species group with maximum node support (Fig. 2; ESM S1), corroborating the view that the *A. autrani* species group is a well-supported natural group that is separated from the other four species groups in this genus (Ottoni et al. 2019). In the present work, we also reaffirm the monophyly of the upper/middle Paraíba do Sul River basin and adjacent drainages clade, which we here term “UMPSAD clade”, and provide molecular diagnostic characters for this clade (Fig. 2). The three species of this clade also possess an exclusive morphological feature, namely in the form of metallic blue or green blotches at the anal-fin base (sometimes also present in the dorsal, pelvic and caudal fins), visible in life and freshly preserved specimens and typically more abundant and conspicuous in larger specimens (see Fig. 1; Ottoni and Costa 2008, fig. 8; Ottoni 2012, fig. 3; Ottoni 2013, figs. 3–6; Ottoni and Schindler 2014; Ottoni et al. 2014). The specimens which we collected from the upper Paranaíba River drainage also feature this characteristic color pattern (Fig. 1c), so that also morphology supports our DNA-based results.

Our combined species delimitation analyses (WP and CBB) revealed the existence of three *Australoheros* species in the UMPSAD clade: *Australoheros barbosae*, *A. macacuensis* and *A. robustus*. Our analyses further suggest that two taxa previously designated as *Australoheros paraibae* and *Australoheros tavaresi* are possible junior synonyms of *A. barbosae*, and that *Australoheros mattosi* is a possible junior synonym of *A. robustus* (ESM S3). *Australoheros montanus*, which also occurs in this drainage and is morphologically distinct, for example in coloration, could not be included here due to the absence of cyt *b* sequence data. However, with at least three species present in the upper/middle Paraíba do Sul River basin, our results challenge the view of Říčan et al. (2011), who considered all *Australoheros* from eastern Brazil as belonging to a single species, *A. facetus*.

The Paranaíba River is the main tributary of the complex of the upper Paraná basin and has the second highest diversity of fish in the Brazilian State of Minas Gerais (Sampaio et al. 2012). Its headwaters are located in the Serra da Mata da Corda, 1,100 m above sea level, and its drainage area encompasses 222,711 km^2^, with ca. 30% in Minas Gerais (Fagundes et al. 2015). Close to its source, the number of streams and monoculture crops are almost equal in occurrence, with much of the former’s waters used for irrigation, and hence the large number of damns in the municipalities around the Alto Paranaíba region. Originally, the vegetation of the biome was composed of scrubs, short trees and bushes. However, within the last decades, agriculture and land use have led to a substitution of most of the biome toward monocultures, grassland and eucalyptus crops (Lahsen et al. 2016; Hofmann et al. 2021). To date, no record exists for *Australoheros* in the Paranaíba River drainage. Therefore, this study represents a distribution extension of the *Australoheros autrani* species group to this river drainage (Paranaíba), as well as a distribution extension for the upper/middle Paraíba do Sul River basin and adjacent drainages clade for *A. barbosae* (Fig. 1a). That the six new sequences from the upper Paranaíba River drainage were identical to most *A. barbosae* haplotypes from different river drainages and systems suggests a rather recent biogeographical expansion of this species between the upper Paraíba do Sul River basin and upper Grande, Tietê and Paranaíba river drainages, of the upper Paraná River. This is also supported by the mismatch analysis (Fig. 3b). There is no evidence of any kind of direct connection between all these drainages, opening up the question whether this species was translocated there. However, since this species is of no economic interest and there is no sign of species introductions in the area, it is more plausible that *A. barbosae* occurs naturally in the upper Paranaíba River drainage. As a result of our work, the geographic distribution of *A. barbosae* should be revised to now include the upper Paraíba do Sul River basin, the upper Rio Grande, the Tietê and the Paranaíba river drainages, of the upper Paraná River basin, whereas the current distribution of *A. macacuensis* is restricted to the Macacu River basin in the Guanabara Bay. The current distribution of *A. robustus* includes the middle Paraíba do Sul River basin, upper Paraopebas, the Rio das Velhas drainages, tributaries of the upper São Francisco River basin, as well as the headwaters border area between the Rio Doce and the São Francisco River basins. All these river systems are located in south-eastern Brazil (Fig. 1a).

The present work highlights, once more, the importance of including molecular data and approaches in taxonomy and species delimitation as well as in biogeographic analyses, as they provide more accurate estimates of biodiversity, especially when done in combination with morphological analyses. By providing a robust taxonomic and phylogeographic framework for the UMPSAD clade of *Australoheros*, our study contributes to a better understanding of the cichlid fauna in an area where cichlids remain understudied. Regarding the other clades within the *Australoheros autrani* species group, the Southern Mata Atlântica clade species is apparently well established in terms of taxonomy. However, for the Northern Mata Atlântica clade, work similar to this one would be needed to accurately estimate its diversity.

## Supplementary Information

Below is the link to the electronic supplementary material.

Supplementary file1 ESM S1 Phylogenetic haplotype tree based on maximum likelihood (ML) (PDF 7414 KB)

Supplementary file2 ESM S2 Results from the mismatch analyses (XLSX 15 KB)

Supplementary file3 ESM S3 Taxonomic decisions based on the combined results of the WP and CBB within the UMPSAD-clade (XLSX 10 KB)
