# Supplementary material for: Species diversity and phylogeography of the Australoheros autrani group (Teleostei, Cichlidae) in eastern Brazil
Source: Ichthyol Res. 2022 Sep 27;70(3):378–85. doi: 10.1007/s10228-022-00888-9 (PMC10293369; doi:10.1007/s10228-022-00888-9)
Supplement: Supplementary file 1 — Supplementary file1 ESM S1 Phylogenetic haplotype tree based on maximum likelihood (ML) (PDF 7414 KB) [file 10228_2022_888_MOESM1_ESM.pdf]

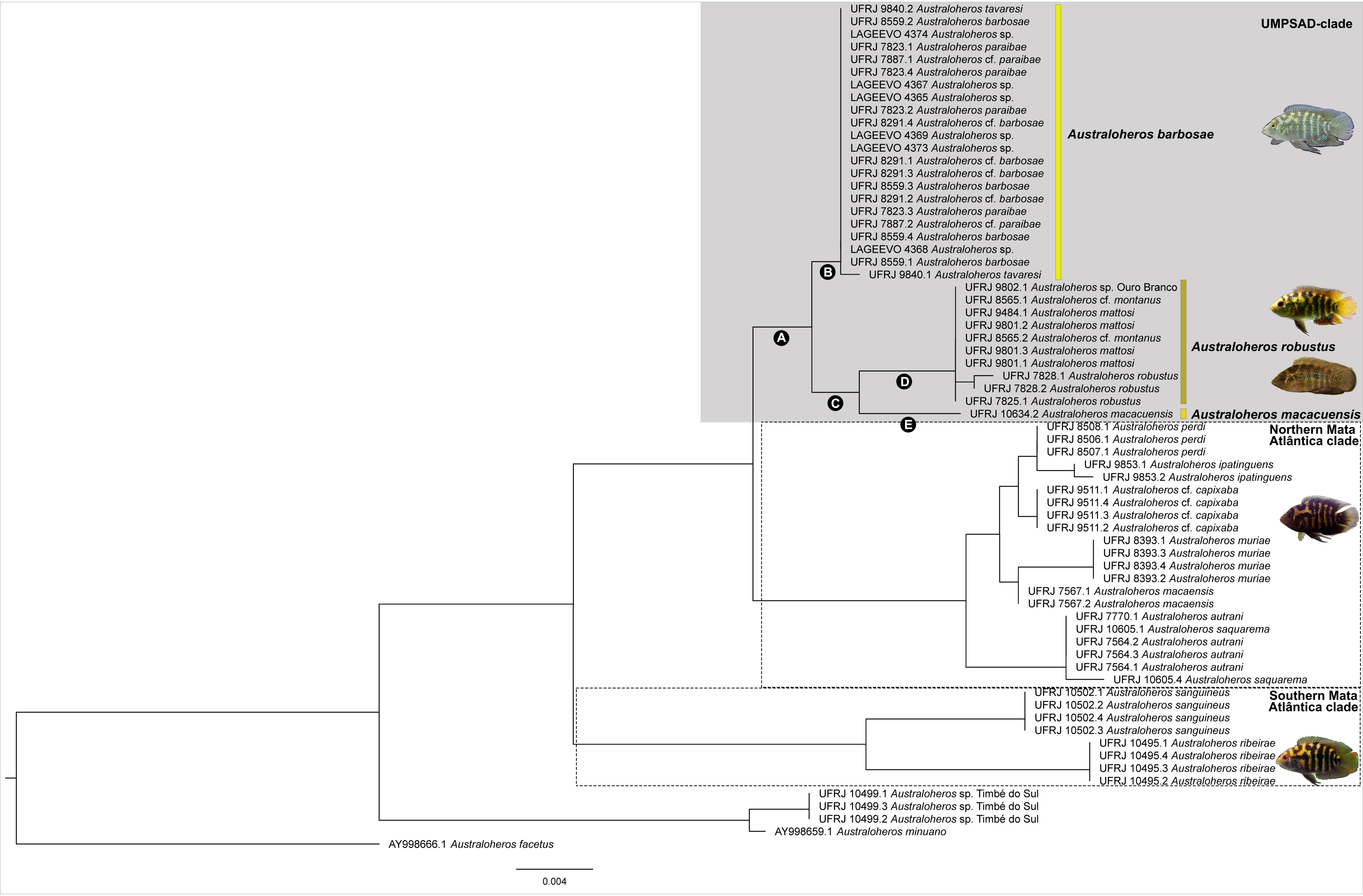
UMPSAD-clade
UFRJ 9840.2 Australoheros tavaresi
UFRJ 8559.2 Australoheros barbosae
LAGEEVO 4374 Australoheros sp.
UFRJ 7823.1 Australoheros paraibae
UFRJ 7887.1 Australoheros cf. paraibae
UFRJ 7823.4 Australoheros paraibae
LAGEEVO 4367 Australoheros sp.
LAGEEVO 4365 Australoheros sp.
UFRJ 7823.2 Australoheros paraibae
UFRJ 8291.4 Australoheros cf. barbosae
LAGEEVO 4369 Australoheros sp.
LAGEEVO 4373 Australoheros sp.
UFRJ 8291.1 Australoheros cf. barbosae
UFRJ 8291.3 Australoheros cf. barbosae
UFRJ 8559.3 Australoheros barbosae
UFRJ 8291.2 Australoheros cf. barbosae
UFRJ 7823.3 Australoheros paraibae
UFRJ 7887.2 Australoheros cf. paraibae
UFRJ 8559.4 Australoheros barbosae
LAGEEVO 4368 Australoheros sp.
UFRJ 8559.1 Australoheros barbosae
UFRJ 9840.1 Australoheros tavaresi
Australoheros barbosae
UFRJ 9802.1 Australoheros sp. Ouro Branco
UFRJ 8565.1 Australoheros cf. montanus
UFRJ 9484.1 Australoheros mattosi
UFRJ 9801.2 Australoheros mattosi
UFRJ 8565.2 Australoheros cf. montanus
UFRJ 9801.3 Australoheros mattosi
UFRJ 9801.1 Australoheros mattosi
UFRJ 7828.1 Australoheros robustus
UFRJ 7828.2 Australoheros robustus
UFRJ 7825.1 Australoheros robustus
Australoheros robustus
UFRJ 10634.2 Australoheros macacuensis
Australoheros macacuensis
A
B
C
D
E
Northern Mata Atlântica clade
UFRJ 8508.1 Australoheros perdi
UFRJ 8506.1 Australoheros perdi
UFRJ 8507.1 Australoheros perdi
UFRJ 9853.1 Australoheros ipatinguens
UFRJ 9853.2 Australoheros ipatinguens
UFRJ 9511.1 Australoheros cf. capixaba
UFRJ 9511.4 Australoheros cf. capixaba
UFRJ 9511.3 Australoheros cf. capixaba
UFRJ 9511.2 Australoheros cf. capixaba
UFRJ 8393.1 Australoheros muriae
UFRJ 8393.3 Australoheros muriae
UFRJ 8393.4 Australoheros muriae
UFRJ 8393.2 Australoheros muriae
UFRJ 7567.1 Australoheros macaensis
UFRJ 7567.2 Australoheros macaensis
UFRJ 7770.1 Australoheros autrani
UFRJ 10605.1 Australoheros saquarema
UFRJ 7564.2 Australoheros autrani
UFRJ 7564.3 Australoheros autrani
UFRJ 7564.1 Australoheros autrani
UFRJ 10605.4 Australoheros saquarema
Southern Mata Atlântica clade
UFRJ 10502.1 Australoheros sanguineus
UFRJ 10502.2 Australoheros sanguineus
UFRJ 10502.4 Australoheros sanguineus
UFRJ 10502.3 Australoheros sanguineus
UFRJ 10495.1 Australoheros ribeirae
UFRJ 10495.4 Australoheros ribeirae
UFRJ 10495.3 Australoheros ribeirae
UFRJ 10495.2 Australoheros ribeirae
UFRJ 10499.1 Australoheros sp. Timbé do Sul
UFRJ 10499.3 Australoheros sp. Timbé do Sul
UFRJ 10499.2 Australoheros sp. Timbé do Sul
AY998659.1 Australoheros minuano
AY998666.1 Australoheros facetus
0.004
